# Plastome Evolution in Saxifragaceae and Multiple Plastid Capture Events Involving *Heuchera* and *Tiarella*

**DOI:** 10.3389/fpls.2020.00361

**Published:** 2020-04-24

**Authors:** Lu-Xian Liu, Ying-Xue Du, Ryan A. Folk, Shen-Yi Wang, Douglas E. Soltis, Fu-De Shang, Pan Li

**Affiliations:** ^1^Key Laboratory of Plant Stress Biology, School of Life Sciences, Henan University, Kaifeng, China; ^2^Department of Biological Sciences, Mississippi State University, Starkville, MS, United States; ^3^Department of Botany, University of Wisconsin-Madison, Madison, WI, United States; ^4^Florida Museum of Natural History, University of Florida, Gainesville, FL, United States; ^5^Department of Biology, University of Florida, Gainesville, FL, United States; ^6^Laboratory of Systematic & Evolutionary Botany and Biodiversity, College of Life Sciences, Zhejiang University, Hangzhou, China

**Keywords:** the *Heuchera* group, Saxifragaceae, plastome, phylogenomics, *Heucherella*

## Abstract

Saxifragaceae, a family of over 600 species and approximately 30 genera of herbaceous perennials, is well-known for intergeneric hybridization. Of the main lineages in this family, the *Heuchera* group represents a valuable model for the analysis of plastid capture and its impact on phylogeny reconstruction. In this study, we investigated plastome evolution across the family, reconstructed the phylogeny of the *Heuchera* group and examined putative plastid capture between *Heuchera* and *Tiarella*. Seven species (11 individuals) representing *Tiarella*, as well as *Mitella* and *Heuchera*, were selected for genome skimming. We assembled the plastomes, and then compared these to six others published for Saxifragaceae; the plastomes were found to be highly similar in overall size, structure, gene order and content. Moreover, *ycf*15 was lost due to pseudogenization and *rpl*2 lost its only intron for all the analyzed plastomes. Comparative plastome analysis revealed that size variations of the plastomes are purely ascribed to the length differences of LSC, SSC, and IRs regions. Using nuclear ITS + ETS and the complete plastome, we fully resolved the species relationships of *Tiarella*, finding that the genus is monophyletic and the Asian species is most closely related to the western North American species. However, the position of the *Heuchera* species was highly incongruent between nuclear and plastid data. Comparisons of nuclear and plastid phylogenies revealed that multiple plastid capture events have occurred between *Heuchera* and *Tiarella*, through putative ancient hybridization. Moreover, we developed numerous molecular markers for *Tiarella* (e.g., plastid hotspot and polymorphic nuclear SSRs), which will be useful for future studies on the population genetics and phylogeography of this disjunct genus.

## Introduction

In the past, studies have relied heavily on organellar markers for inferring phylogenetic relationships. However, chloroplast and mitochondrial genes often show markedly different phylogenetic patterns from nuclear markers (Rieseberg and Soltis, 1991; Toews and Brelsford, 2012). Various factors, including convergent evolution, lineage sorting, hybridization and introgression, may cause phylogenetic incongruence between nuclear and plastid DNA (e.g., Soltis and Kuzoff, 1995; McKinnon et al., 2001; Acosta and Premoli, 2010). Incomplete lineage sorting and hybridization/introgression are particularly hard to distinguish due to their similar phylogenetic signatures (Wendel and Doyle, 1998). However, hybridization is now more readily detected due to advances in statistical methods and data collection strategies (Joly et al., 2009; Folk et al., 2018). One extreme result of hybridization is plastid capture, in which the cytoplasm of one species is replaced by that of another species through inter-species hybridization and subsequent backcrossing, yielding a plant with a novel combination of nuclear and plastid genomes (Rieseberg and Soltis, 1991). Plastid capture has been reported across numerous plant lineages (Okuyama et al., 2005; Fehrer et al., 2007; Acosta and Premoli, 2010; Gurushidze et al., 2010; Mir et al., 2010).

Saxifragaceae, a family of over 600 species and approximately 30 genera of herbaceous perennials, are found mostly in the Northern Hemisphere with centers of diversity in the Himalayas, East Asia, and Western North America (Deng et al., 2015). Due to factors such as morphological stasis, convergent morphological evolution, and disjunct distributions, relationships within Saxifragaceae have historically confused botanists (Engler, 1930; Morgan and Soltis, 1993). The family comprises two main lineages: Saxifragoids, which include *Saxifraga* L. and *Saxifragella* Engl., and Heucheroids, which include all other genera. The Heucheroids are noted for examples of intergeneric hybridization. For example, nine genera of herbaceous perennials (*Bensoniella* C.V. Morton, *Conimitella* Rydb., *Elmera* Rydb., *Tellima* R. Br., *Tolmiea* Torr. et A. Gray, *Tiarella* L., *Lithophragma* (Nutt.) Torr. et A. Gray, *Mitella* L., and *Heuchera* L.) comprise the *Heuchera* group, a particularly valuable model for the analysis of plastid capture and its impact on phylogeny reconstruction due to evidence of numerous hybridization events among and within those genera (Soltis et al., 1991; Soltis and Kuzoff, 1995).

Within the *Heuchera* group, *Mitella* and *Heuchera* are the two largest genera, consisting of 21 and 43 currently recognized species, respectively (Folk and Freudenstein, 2014; Okuyama, 2015). *Lithophragma* and *Tiarella* comprise nine and three species, respectively. *Tolmiea* (2 spp.) and the remaining four monotypic genera (*Bensoniella*, *Conimitella*, *Elmera*, and *Tellima*) are solely distributed in western North America. Six of these genera are known to hybridize (Soltis and Bohm, 1985; Soltis and Soltis, 1986; Soltis et al., 1991), with intergeneric hybrids being reported between *Heuchera* and *Tiarella* (Spongberg, 1972), *Tellima* and *Tolmiea* (Soltis and Bohm, 1985), and *Mitella* and *Conimitella* (Soltis and Soltis, 1986). Numerous interspecific hybrids have been reported within *Heuchera* (Rosendahl et al., 1936). Several studies have reported that plastid capture likely occurred between species of *Heuchera*, as well as between *Tellima* and *Mitella* and between *Heuchera* and *Mitella.* Likewise, there is evidence for gene flow between *Tiarella* and *Heuchera* (Soltis, 1991; Soltis et al., 1991; Folk et al., 2017).

*Tiarella* is a small genus within the *Heuchera* group and contains only three species: *Tiarella polyphylla* D. Don, *Tiarella cordifolia* L., and *Tiarella trifoliata* L. (Wu and Raven, 2001; Wells and Elvander, 2009). The genus shows an interesting eastern Asia (*T. polyphylla*)-western North America (*T. trifoliata*)-eastern North America (*T. cordifolia*) disjunct distribution pattern, with one species each in these three areas. Soltis (1991) resolved *T. trifoliata* and *T. cordifolia* as paraphyletic to *Heuchera* as well as some other members of the *Heuchera* group based on restriction site analysis of chloroplast (cp) DNA (*T. polyphylla* was not sampled in this study). Subsequently, Soltis and Kuzoff (1995) revealed a clade containing *T. trifoliata* and *T. cordifolia* based on nrITS-1 (nuclear internal transcribed spacer) and nrITS-2 sequences. The monophyly of *Tiarella* was confirmed using ITS data by Xiang et al. (1998), who resolved the Asian species as sister to the two North American species. However, Okuyama et al. (2008) revealed that *T. polyphylla* and *T. cordifolia* formed a clade with moderate support and was sister to *T. trifoliata*. Recently, the ancestral area of this genus was found to be western North America as inferred by S-DIVA based on cpDNA + nrDNA data (Deng et al., 2015). However, the phylogenetic relationships among *T. trifoliata*, *T. cordifolia*, and *T. polyphylla* are not yet well resolved and these previous studies have lacked the population-level sampling needed for robust inference of hybridization histories.

High-throughput sequencing technologies have revolutionized the ease with which genomic data can be acquired for any plant species, from angiosperms to bryophytes, regardless of model species or non-model species (Dodsworth, 2015). Advances in sequencing technology has impacted biodiversity science, including systematics, population genetics, DNA barcoding and ecological investigations. Genome skimming sequencing, which involves random sampling of a small percentage of total genomic DNA (gDNA), was first identified by Straub et al. (2012) as a straightforward way to obtain genome-scale data with minimal lab processing. It is a simpler method compared to RNA-seq and RAD-seq, and the high-copy fraction of the genome (plastome, mitogenome, and repetitive nuclear elements) can be deeply sequenced through shallow sequencing of total gDNA. The nrITS and nrETS, various plastid markers and polymorphic nuclear simple sequence repeats (nSSRs) are commonly acquired using this technique (Liu et al., 2018a). This approach has been widely used at different taxonomic levels, for intraspecific “ultra-barcoding” (Kane et al., 2012), intergeneric (McPherson et al., 2013; Li et al., 2017; Liu et al., 2018b), and family level or above phylogenomic analyses (Besnard et al., 2013; Malé et al., 2015; Liu et al., 2017; Xu et al., 2018).

Here, we used a genome skimming approach to investigate the phylogeny of *Tiarella* and test the hypothesis of plastid capture between *Tiarella* and *Heuchera*. Seven species, in total 11 individuals, were selected for genome skimming. We specifically aimed to: (1) assemble, characterize and compare the plastomes among representatives of Saxifragaceae to gain insights into evolutionary patterns; (2) acquire nrITS, nrETS, and plastome data to resolve the phylogeny of *Tiarella* and the direction of plastid capture between *Tiarella* and *Heuchera*; (3) develop and screen appropriate intrageneric markers for *Tiarella*, such as plastid hotspot regions and polymorphic nSSRs.

## Materials and Methods

### Taxon Sampling

Seven species from the *Heuchera* group (11 individuals) including *T. polyphylla* (4), *T. cordifolia* (2), *T. trifoliata* (1), *Heuchera richardsonii* R. Br. (1), *Heuchera villosa* Michx. (1), *Mitella diphylla* L. (1), and *Mitella formosana* (Hayata) Masam. (1) were selected for genome skimming (Supplementary Table S1). Fresh healthy leaves were collected in the field and immediately dried with silica gel for later DNA isolation. Voucher specimens are deposited at the Herbarium of Zhejiang University (HZU).

For broader comparisons, the complete plastomes of *Heuchera parviflora* Bartl (GenBank accession number: KR478645; Folk et al., 2015), as well as the following Saxifragaceae not in the *Heuchera* group were downloaded from GenBank: *Bergenia scopulosa* T.P. Wang (KY412195; Bai et al., 2018), *Mukdenia rossii* (Oliv.) Koidz (MG470844; Liu et al., 2018a), *Oresitrophe rupifraga* Bunge (MF774190 and MG470845; Liu et al., 2018a), and *Saxifraga stolonifera* Curtis (MH191389; Dong et al., 2018).

### DNA Preparation and Sequencing

Total DNA was extracted using Plant DNAzol Reagent (LifeFeng, Shanghai) according to the manufacturer’s protocol with ∼2 mg of the silica-dried leaf tissue. The high molecular weight DNA was sheared using a Covaris S220-DNA Sonicator (Covaris, Inc., Woburn, MA, United States), yielding fragments ≤800 bp. The fragmentation quality was checked on an Agilent Bioanalyzer 2100 (Agilent Technologies). Libraries with an insert size of ∼500 bp were sequenced on an Illumina HiSeq 2500 (paired-end, 150 bp reads) by Beijing Genomics Institute (Shenzhen, China).

### Genome Assembly and Annotation

The raw reads were first filtered by quality with average Phred score < 30 (0.001 error probability). Subsequently, the remaining high-quality sequences were assembled into contigs using the CLC Genomics Workbench v12.0.3 (CLC Inc., Aarhus, Denmark) with the following optimized parameters: deletion and insertion costs of 3, mismatch cost of 2, minimum contig length of 200, bubble size of 98, length fraction, and similarity fraction of 0.9. All resultant contigs were mapped to the reference plastome (*H. parviflora*) using BLAST (NCBI BLAST v2.2.31) and the mapped contigs were ordered and oriented according to the reference genome (Folk et al., 2015). The draft genome was constructed by connecting overlapping terminal sequences, and the plastome was finalized by re-mapping cleaned reads to the draft genome.

**FIGURE 1:**
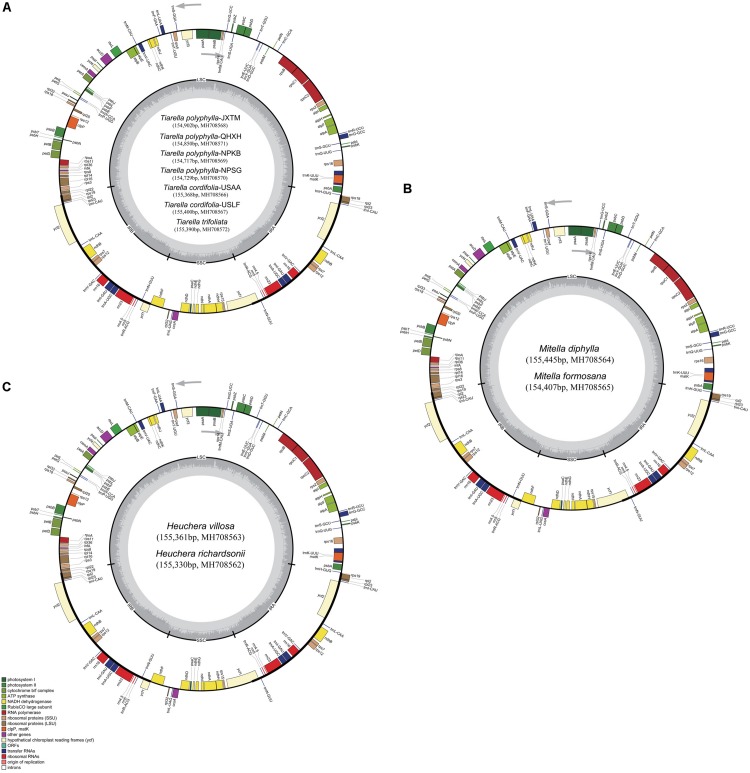
Plastome maps of *Tiarella, Heuchera*, and *Mitella*. **(A)** Seven *Tiarella* plastomes including four *T. polyphylla* individuals, two *T. cordifolia*, and one *T. trifoliata*, **(B)** Two *Mitella* plastomes including *M. diphylla* and *M. formosana* **(C)** Two *Heuchera* plastomes including *H. villosa* and *H. richardsonii*. Genes inside the circle are transcribed clockwise, gene outside are transcribed counter-clockwise. The light gray inner circle corresponds to the AT content, the dark gray to the GC content. Genes belonging to different functional groups are shown in different colors; see the legend for groups.

Complete plastome annotation was performed through the online program Dual Organellar GenoMe Annotator (DOGMA; Wyman et al., 2004). The initial annotation was subsequently inspected and adjusted manually comparing with the reference genome sequence in order to confirm the start and stop codons and the exon-intron boundaries of genes. The tRNA genes were verified using tRNAscan-SE v1.21 (Schattner et al., 2005) with default settings. All the plastome sequences were deposited in GenBank (Figure 1) and the circular gene maps were drawn by the OrganellarGenomeDRAW tool (OGDRAW v1.3.1; Lohse et al., 2013) followed by manual modification.

### Comparison of Complete Plastomes in Saxifragaceae

To compare the sequence variation within the Saxifragaceae, we combined publicly available plastomes of *B. scopulosa*, *M. rossii*, *O. rupifraga* (representing the *Darmera* group of genera; Soltis et al., 2001), and *S. stolonifera*. Multiple sequence alignments of the 12 Saxifragaceae species were performed in MAFFT v7.017 (Katoh and Standley, 2013) under standard parameters, and visually inspected and manually adjusted in GENEIOUS v8.1.7 (Kearse et al., 2012). The sequence identity of the 12 Saxifragaceae plastomes was plotted using the mVISTA program with LAGAN mode (Frazer et al., 2004). Plastid DNA rearrangement analyses of the 12 Saxifragaceae plastomes were performed via whole genome alignment in Mauve v2.3.1 (Darling et al., 2004).

### Sequence Divergence Analysis and Polymorphic nSSR Development for *Tiarella*

To screen variable characters within the genus *Tiarella*, multiple alignments of the seven plastomes of *Tiarella* individuals were carried out using MAFFT v7.017. The average number of nucleotide different (*K*) and total number of mutations (*Eta*) was determined to analyze nucleotide diversity (π) using DnaSP v5.0 (Librado and Rozas, 2009).

In addition, the software CandiSSR v20170602 (Xia et al., 2015) was used to identify candidate polymorphic nSSRs within the genus *Tiarella* based on multiple assembled sequences. Plastid and mitochondrial contigs of the *Tiarella* species were removed from the assembled sequences using NCBI BLAST (v2.2.31) with the plastid (KR478645) and mitochondrial (KR559021) genomes of *H. parviflora* as references. The parameters implemented in CandiSSR are as follows: the flanking sequence length of 100, blast *e*-value cutoff of 1e-10, blast identity cutoff of 95, blast coverage cutoff of 95. Primers were automatically designed in the pipeline based on the Primer3 package (Untergasser et al., 2012) for each target SSRs.

### Acquisition of nrITS and nrETS Sequences

To assemble the nrITS and nrETS sequences of all the accessions (including outgroup, in total nine species, 14 individuals), we downloaded the nrITS and nrETS sequences of *H. parviflora* (GenBank accession number: KM496213 for nrITS and KM496002 for nrETS) from GenBank as references. The assembled contigs were mapped to the reference sequences using BLAST search, and the mapped contigs with the highest score were aligned with the reference sequences again. Then, nrITS and nrETS sequences for all the accessions were extracted from the alignment files. To validate the accuracy of the nrITS and nrETS sequences we acquired, the ITS and ETS sequences from each *Tiarella* species were verified by PCR amplification and Sanger sequencing using the newly developed specific primers (nrITS F: CTCGCCGTT ACTAAGGGAATC, R: CGTAACAAGGTTTCCGTAGGTG; nrETS F: AGCCATTCGCAGTTTCACAG, R: TGGGACC TTGTGCTACACTTTG).

### Phylogenetic Inference

For phylogenetic inference, Maximum likelihood (ML) and Bayesian inference (BI) analyses were performed for the three different datasets: (1) complete plastome sequences; (2) a set of 77 protein coding sequences (CDS) shared by the plastomes (3) nrDNA sequence data including ITS and ETS. For plastid data, the ingroup included 81 accessions from the genera *Tiarella*, *Heuchera*, *Bensoniella*, and *Mitella*. These sequences included 11 accessions of seven taxa sequenced in this study, one accession downloaded from GenBank (*H. parviflora*, the GenBank accession number: KR478645), and the alignment data file containing 69 plastomes from Dryad (doi: https://doi.org/10.5061/dryad.cd546; published by Folk et al., 2017). For nrDNA data, the ingroup included 65 accessions from the same four genera, which consisted of 11 accessions sequenced in this study, as well as other 54 accessions downloaded from GenBank (Supplementary Table S2). The species used in the plastid and nrDNA datasets were kept largely consistent, except for some species for which nrDNA data were not available. One individual of *M. rossii* and two individuals of *O. rupifraga* were selected as outgroups based on Deng et al. (2015).

First, we used PARTITIONFINDER v2.1.1 (Lanfear et al., 2017) to determine the optimal data partition scheme and nucleotide substitution models for the two alignments according to the Akaike information criterion (AICc), resulting in three partitions for the 77 CDS regions (the first, second and third codon positions) and four partitions for nrDNA (ETS, ITS1, 5.8s rRNA and ITS2) (Table 1). The software jModelTest v2.1.4 (Posada, 2008) was used to determine the best-fit nucleotide substitution model (GTR + I + G) for the complete plastome sequences. ML analyses were implemented in RAxML-HPC v8.1.11 on the CIPRES cluster (Miller et al., 2010) using the optimal partitioning scheme and substitution model. 1000 bootstrap iterations were conducted with other parameters using the default settings. BI analyses were performed in MrBayes v3.2.3 (Ronquist and Huelsenbeck, 2003) using the same model selection criteria for two datasets. The Markov chain Monte Carlo (MCMC) algorithm was run with two independent chains with a random starting tree and default priors for 5,000,000 generations, with every 1000 generations for trees sampling. Convergence of the MCMC chains was assumed when the average standard deviation of split frequencies reached 0.01 or less.

**TABLE 1:** Best partitioning scheme and nucleotide substitution models for nrDNA and 77 CDS data determined by PartitionFinder v2.1.1.

Data type	Subset	Partition names	Best model
nrDNA	1	ETS	HKY + G
	2	ITS1, ITS2	SYM + G
	3	5.8s rRNA	K80
77 CDS	1	cpCDS_1stpos	GTR + I + G
	2	cpCDS_2stpos	GTR + I + G
	3	cpCDS_3stpos	GTR + I + G

*The models of evolution include gamma distributed rate variation among sites (G) and the proportion of invariable sites (I).*

## Results

### Genome Organization and Features

We generated paired-end clean reads ranging from 15,431,294 for *T. polyphylla*-NPSG to 49,735,470 for *M. diphylla*, respectively (Table 2). After filtering by quality with average Phred score < 30, the average length of remaining paired-end reads ranged from 100.43 bp (*T. polyphylla*-NPKB) to 135.53 bp (*H. villosa*), and the number of reads were between 13,331,090 (*T. polyphylla*-NPSG) and 44,127,123 (*M. diphylla*). The number of *de novo* assembled contigs from genome skimming data ranged from 315,998 (*T. trifoliata*) to 562,337 (*M. diphylla*). Each draft plastome of all the 11 accessions was generated from three initial contigs corresponding to the LSC, SSC and IR_*B*_/IR_*A*_ regions, with no gaps or undetermined sites. The nucleotide coverage at each site for final plastome reconstruction was ranged from 11 (*T. polyphylla*-NPSG) to 5996 (*H. richardsonii*), and the average nucleotide coverage was ranged from 159 (*T. polyphylla*-NPSG) to 4682 (*H. richardsonii*), respectively (Table 2). Eventually, we found that each draft plastome sequence was absolutely identical with the corresponding final genome sequence.

**TABLE 2:** Summary of the 11 individuals from the *Heuchera* group sequenced in this study.

	*T. polyphylla* (JXTM)	*T. polyphylla* (NPKB)	*T. polyphylla* (NPSG)	*T. polyphylla* (QHXH)	*T. cordifolia* (USAA)	*T. cordifolia* (USLF)	*T. trifoliata*	*M. diphylla*	*M. formosana*	*H. richardsonii*	*H. villosa*
Number of reads	19,350,774	19,945,556	15,431,294	28,464,000	17,188,180	18,746,754	17,640,500	49,735,470	17,440,054	35,240,056	35,072,040
Number of reads after trim	16,090,346	17,241,720	13,331,090	23,386,919	14,711,553	14,129,448	15,338,179	44,127,123	15,159,154	31,288,357	30,888,194
Average length after trim (bp)	125.04	100.43	102.02	126.61	129.11	108.51	134.73	132.58	129.58	135.50	135.53
Minimal nucleotide coverage (×)	54	13	11	72	21	54	28	17	18	70	64
Maximal nucleotide coverage (×)	1675	289	217	3110	1254	1684	1174	3534	649	5996	4856
Average nucleotide coverage (×)	1263	221	159	2534	935	717	727	1960	493	4682	3733
Total plastid DNA size	154,902	154,717	154,729	154,850	155,368	155,400	155,390	155,445	154,407	155,330	154,850
Length of large single copy (LSC) region	86,088	85,892	85,914	86,035	86,086	86,056	86,149	86,242	85,506	86,060	86,058
Length of inverted repeat (IR) region	25,396	25,388	25,391	25,397	25,633	25,636	25,622	25,631	25,608	25,633	25,633
Length of small single copy (SSC) region	18,022	18,049	18,033	18,021	18,046	18,072	17.997	17,941	17,685	18,004	18,037
Total GC content (%)	37.70	37.70	37.80	37.80	37.80	37.80	37.80	37.80	37.90	37.80	37.80
LSC	35.80	35.80	35.80	35.80	35.80	35.80	35.80	35.80	36.00	35.80	35.80
IR	43.10	43.10	43.10	43.10	43.10	43.10	43.10	43.10	43.10	43.10	43.10
SSC	32.10	32.10	32.10	32.10	32.20	32.10	32.10	32.20	32.30	32.20	32.20
Total number of genes	113	113	113	113	113	113	113	113	113	113	113
Protein encoding	79	79	79	79	79	79	79	79	79	79	79
tRNA	30	30	30	30	30	30	30	30	30	30	30
rRNA	4	4	4	4	4	4	4	4	4	4	4
Number of genes duplicated in IR	19	19	19	19	19	19	19	19	19	19	19

All the plastomes sequenced in this study had a standard angiosperm structure comprising two copies of the IR region (25,388–25,636 bp) separated by the LSC region (85,506–86,242 bp) and SSC region (17,685–18,072 bp; Figure 1 and Table 2). For *Tiarella* species, the complete plastomes ranged from 154,717 bp in *T. polyphylla*-NPKB to 155,400 bp in *T. cordifolia*-USLF (Table 2). The overall GC content ranged narrowly from 37.70% to 37.80%, whereas the GC content in the LSC, SSC and IR regions were 35.80%, 32.10–32.20%, and 43.10%, respectively. For *Heuchera* and *Mitella* species, the complete plastomes ranged from 154,407 bp in *M. formosana* to 155,330 bp in *H. richardsonii* (Table 2). The overall GC content ranged from 37.80% to 37.90%, whereas the GC content in the LSC, SSC, and IR regions were 35.80–36.00%, 32.20–32.30%, and 43.10%, respectively.

The plastomes of all 11 accessions encoded an identical set of 132 genes, of which 113 were unique and 19 were duplicated in the IR regions (Table 2). Among the 113 unique genes, there were 79 protein-coding genes, 30 tRNA genes and four rRNA genes (Table 3). Six tRNA genes and eight protein-coding genes contained single intron, and three genes including *rps*12, *clp*P, and *ycf*3 contained two introns. The 5′-end exon of the *rps*12 gene was located in the LSC region, and the intron and 3′-end exon of the gene were situated in the IR region.

**TABLE 3:** Genes contained in Saxifragaceae plastomes (113 genes in total).

Category	Group of gene	Name of gene			
Self-replication	Ribosomal RNA genes	*rrn*4.5^*a*^	*rrn*5^*a*^	*rrn*16^*a*^	*rrn*23^*a*^
	Transfer RNA genes	*trnA*-UGC^a^* *trnF-*GAA *trnH-*GUG *trnL-*CAA^*a*^ *trnN-*GUU^*a*^ *trnR-*UCU *trnT-*GGU *trnW-*CCA	*trnC-*GCA *trnfM*-CAU *trnI-*CAU^*a*^ *trnL-*UAA* *trnP-*UGG *trnS-*GCU *trnT-*UGU *trnY-*GUA	*trnD-*GUC *trnG-*GCC* *trnI-*GAU^a^* *trnL-*UAG *trnQ-*UUG *trnS-*GGA *trnV-*GAC^*a*^	*trnE-*UUC *trnG-*UCC *trnK-*UUU* *trnM-*CAU *trnR-*ACG^*a*^ *trnS-*UGA *trnV-*UAC*
	Small subunit of ribosome	*rps*2 *rps8 rps*15	*rps*3 *rps*11 *rps*16*	*rps*4 *rps*12^a,b^** *rps*18	*rps*7^*a*^ *rps*14 *rps*19^a^
	Large subunit of ribosome	*rpl*2^*a*^ *rpl*22 *rpl*36	*rpl*14 *rpl*23^*a*^	*rpl*16* *rpl*32	*rpl*20 *rpl*33
	RNA polymerase subunits	*rpo*A	*rpo*B	*rpo*C1*	*rpo*C2
Photosynthesis	Subunits of photosystem I	*psa*A *psa*J	*psa*B *ycf*3**	*psa*C	*psa*I
	Subunits of photosystem II	*psb*A *psb*E *psb*J *psb*N	*psb*B *psb*F *psb*K *psb*T	*psb*C *psb*H *psb*L *psb*Z	*psb*D *psb*I *psb*M
	Subunits of cytochrome	*pet*A *pet*L	*pet*B* *pet*N	*pet*D*	*pet*G
	Subunits of ATP synthase	*atp*A *atp*H	*atp*B *atp*I	*atp*E	*atp*F*
	Large subunit of Rubisco	*rbc*L			
	Subunits of NADH Dehydrogenase	*ndh*A* *ndh*E *ndh*I	*ndh*B^a^* *ndh*F *ndh*J	*ndh*C *ndh*G *ndh*K	*ndh*D *ndh*H
Other genes	Translational initiation factor	*inf*A			
	Maturase	*mat*K			
	Envelope membrane protein	*cem*A			
	Subunit of acetyl-CoA	*acc*D			
	C-type cytochrome synthesis gene	*ccs*A			
	Protease	*clp*P**			
Unknown function	Conserved open reading frames	*ycf*1^*a*^ (part)	*ycf*2^*a*^	*ycf*4	

*^*a*^Two gene copies in IRs; ^*b*^gene divided into two independent transcription units; one and two asterisks indicate one- and two-intron containing genes, respectively.*

### Comparisons of the Plastomes in Saxifragaceae

**FIGURE 2:**
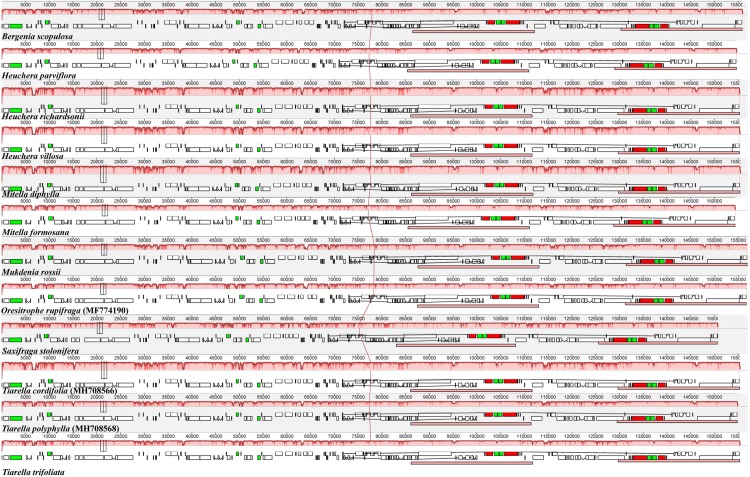
Mauve alignment of 12 Saxifragaceae plastomes. Within each of the alignment, local collinear blocks are represented by blocks of the same color connected by lines.

There were seventeen complete Saxifragaceae plastome sequences available in GenBank, including the 11 genomes sequenced in this study. We selected 12 of them representing different species to compare their structural organization. The 12 Saxifragaceae plastomes exhibited high levels of sequence similarity and structural conservation (Figure 2), and no rearrangement occurred in gene organization after verification. IR regions were more conservative than the LSC and SSC regions (Supplementary Figure S1).

**FIGURE 3:**
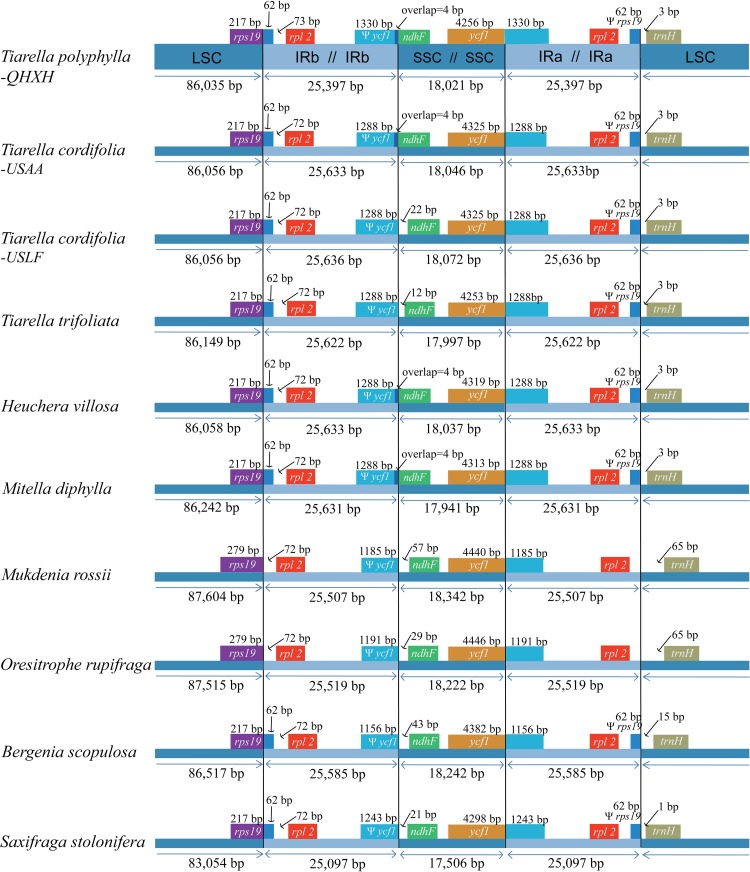
Comparison of boundaries of the large single-copy (LSC), small single-copy (SSC), and inverted repeat (IR) regions among the ten Saxifragaceae plastomes. We show only *Heuchera villosa* and *Mitella diphylla* as representative of identical boundary types in these genera. For the location of the two inverted repeat regions (IR_*A*_ and IR_*B*_), refer to Figure 1.

Among the 12 Saxifragaceae species, the plastome size ranged from 150,752 to 156,960 bp in length. *S. stolonifera* exhibited the smallest genome size, while *M. rossii* had the largest genome size. The plastome size was typically between 154,000 bp and 157,000 bp. Size variation in Saxifragaceae plastomes is attributable to the length differences of LSC, SSC, and IRs regions (Figure 3). We compared the exact IR border positions and their adjacent genes between the Saxifragaceae plastomes (Figure 3). The genes *rps*19-*rpl*2-*trn*H and *ycf*1-*ndh*F were located in the junctions of LSC/IR and SSC/IR regions. The *ycf*1 gene spanned the SSC/IR_*A*_ region and the pseudogene fragment of ^ψ^ *ycf*1 varies from 1156 to 1330 bp. The *ndh*F gene shared four nucleotides with the ^ψ^ *ycf*1 in *T. polyphylla*, *T. cordifolia*-USAA, three *Heuchera* and two *Mitella* species, but was separated from ^ψ^ *ycf*1 by a spacer with the length ranging from 12 to 57 bp in the rest species. The *rps*19 gene did not extend to the IR_*B*_ region in *M. rossii* and *O. rupifraga*, but crossed the LSC/IR_*B*_ region with 62 bp located at the IR_*B*_ region in the other Saxifragaceae species. The *rpl*2 gene was separated from the LSC/IR_*B*_ border by a spacer varied from 72 to 135 bp, as well as the *trn*H gene was separated from the IR_*A*_/LSC border by a spacer varied from 1 to 65 bp.

The *ycf*15 gene, which was represented only by a small open reading frame (ORF), appeared to have been lost from all species in the family via pseudogenization, while the *inf*A gene, commonly lost across the angiosperms, was always present. Likewise, the *rpl*2 gene was missing its single intron in all Saxifragaceae species investigated here. Therefore, the loss of *ycf*15 and the *rpl*2 intron may represent two synapomorphies for Saxifragaceae (Downie et al., 1991; Dong et al., 2013).

### Sequence Divergence Analysis and Polymorphic nSSR Development for *Tiarella*

**FIGURE 4:**
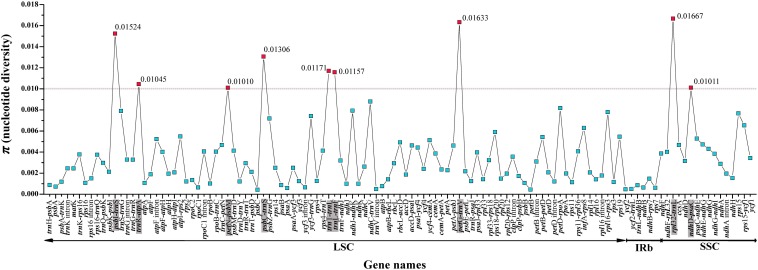
Comparative analysis of the nucleotide variability (π) values within the genus *Tiarella*. The dotted line represents π > 0.01; genes surpassing this threshold are highlighted in gray.

For the entire plastome, the IR regions of Saxifragaceae plastomes (nucleotide diversity, π = 0.00024) showed more conserved than either the LSC (π = 0.00237) or the SSC (π = 0.00387) regions. We compared coding genes, non-coding regions and intron regions among seven individuals of the three *Tiarella* species to find divergence hotspots, generating 119 loci (46 coding genes, 61 intergenic spacers, and 12 intron regions) (Figure 4). For the 119 regions, the π value for each locus ranged from 0.00042 (*psb*C) to 0.01667 (*rpl*32-*trn*L). We found nine highly divergent regions (π > 0.01): *psb*I-*trn*S (0.01524), *trn*R-*atp*A (0.01045), *pet*N-*psb*M (0.0101), *psb*C-*trn*S (0.01306), *trn*T-*trn*L (0.01171), *trn*L-*trn*F (0.01157), *pet*G-*trn*W (0.01633), *rpl*32-*trn*L (0.01667), and *ndh*D-*psa*C (0.01011). These levels of intrageneric variation suggested the suitability of these regions as markers for the genus *Tiarella*.

**FIGURE 5:**
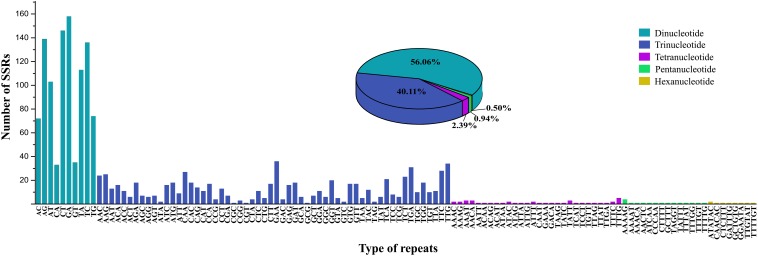
The distribution information of the polymorphic nuclear simple sequence repeats (nSSRs) within the genus *Tiarella*. The pie chart shows the overall prevalence of repeat lengths; the histogram shows the frequency distribution of prevalent repeat classes.

A total of 2,600 candidate polymorphic nSSRs were identified within the genus *Tiarella*. After removing the loci with the sequence similarity <90% (211) and no available primers designed (589), we obtained 1,800 polymorphic nSSRs with the standard deviation ranged from 0.40 to 4.97 for this genus (Supplementary Table S2). Among them, di- (1,009), tri- (722), tetra- (43), penta- (17), and hexanucleotides (9) accounted for 56.06%, 40.11%, 2.39%, 0.94%, and 0.50%, respectively (Figure 5). With regard to these nSSRs, GA (158) of di-, GAA (36) of tri-, TTTG (5) of tetra-, AAAAG (4) of penta-, and ATATAC (2) of hexanucleotides occupied the predominant proportion.

### Phylogenetic Inference

For the nuclear dataset, we obtained the nrITS and nrETS regions of the fourteen species sequenced here and supplemented this matrix with data from GenBank (primarily from Okuyama et al., 2008; Folk and Freudenstein, 2014). The nrITS regions newly sequenced in this study ranged from 631 bp in *T. polyphylla*-NPKB to 656 bp in *M. diphylla*. The length of the nrETS region varied slightly from 457 bp in *O. rupifraga* to 466 bp in *H. richardsonii* and *M. diphylla*. These sequences have been deposited in GenBank (Supplementary Table S3). Sequences for nrITS and nrETS regions of *Tiarella* obtained using Sanger sequencing are identical to those we obtained from genome skimming data. The concatenated matrix of nrITS and nrETS comprised 68 taxa with an aligned length of 1,078 bp. The aligned lengths of the complete plastome and 77-CDS datasets (in total 84 individuals) was 163,155 bp and 67,806 bp, respectively.

**FIGURE 6:**
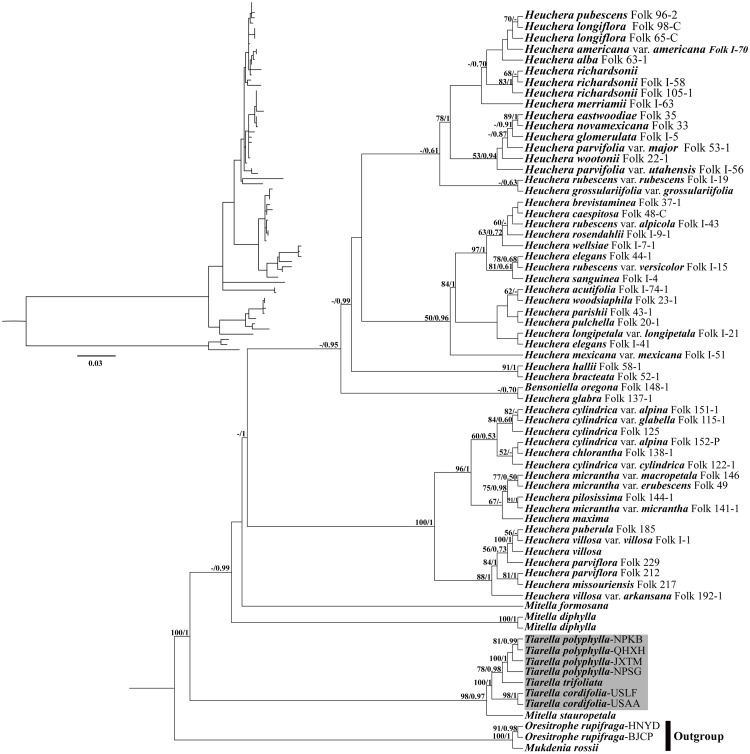
Phylogenetic tree reconstruction using maximum likelihood (ML) based on ITS + ETS sequences. The inset topology in the upper left shows the relative branch lengths in per-site substitutions. Numbers above the branches represent ML bootstrap/Bayesian posterior probability (BS/PP). Hyphens indicate a bootstrap value or posterior probability <50%.

For the analyses with nrDNA data, the tree topologies recovered from ML and BI analyses were congruent with one another (Figure 6). The monophyly of *Tiarella* had maximal support (reported hereafter as ML bootstrap support (BS)/BI posterior probability (PP); BS/PP = 100/1). Within *Tiarella*, the four individuals of *T. polyphylla* (BS/PP = 100/1) and two individuals of *T. cordifolia* (BS/PP = 98/1) were resolved as reciprocally monophyletic, and we obtained decisive support for the topology [(*T. polyphylla*, *T. trifoliata*), *T. cordifolia*]. *Heuchera* was also monophyletic with full support in BI analysis (PP = 1) when including the monotypic genus *Bensoniella* (to date this placement of *Bensoniella* has been seen only in ribosomal DNA data; Okuyama et al., 2008; Folk and Freudenstein, 2014). In addition, the three *Mitella* species investigated were found to be polyphyletic. Of them, *Mitella stauropetala* Piper was sister to the *Tiarella* clade with high support (BS/PP = 98/0.97), while the other two *Mitella* species were clustered with the *Heuchera* clade. *Mitella formosana* was weakly supported to be sister to the *Heuchera* clade (BS/PP < 50/0.5), followed by *M. diphylla*, which is subsequently sister to the above clade (BS < 50%, PP = 0.99).

**FIGURE 7:**
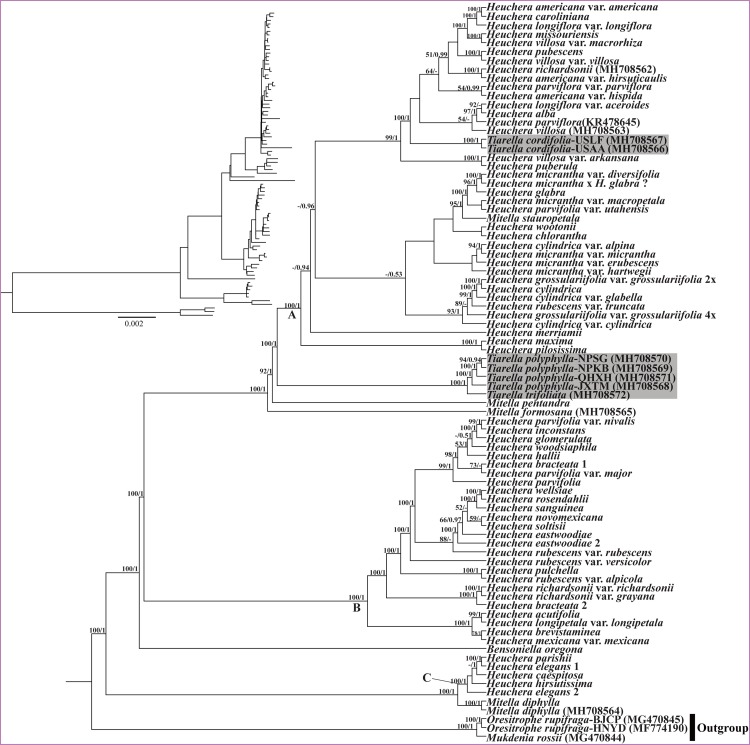
Phylogenetic tree reconstruction using maximum likelihood (ML) based on whole plastome sequences. The inset topology in the upper left shows the relative branch lengths in per-site substitutions. Numbers above the branches represent ML bootstrap/Bayesian posterior probability (BS/PP). Hyphen indicate a bootstrap value or posterior probability <50%. Clade labels follow those for chloroplast clades recovered in Folk et al. (2017). Gray highlighted labels indicate the position of *Tiarella* plastomes.

For the analyses of plastid data, the ML and BI trees were identical in topology within each data matrix for the plastomes (Figure 7) and the 77 CDS regions (Supplementary Figure S2). Between datasets, the results were generally consistent; support values were generally greater in the plastid tree than for the 77-CDS tree, probably due to the inclusion of more informative loci. However, the results based on plastid data are strongly incongruent with those based on nrDNA data. The *Heuchera* species were divided into three major clades (labeled clades A, B, C in Figure 7), all with full support (BS/PP = 100/1). *M. stauropetala* and the two *T. cordifolia* individuals were nested deeply within *Heuchera* plastid Clade A. The other two *Tiarella* species formed a clade sister to *Heuchera* plastid Clade A; they subsequently formed a clade with *M. pentandra* Hook. and *M. formosana*. Clade B entirely comprised most of the remaining *Heuchera* species, including 26 accessions from 20 species. For the third *Heuchera* plastid clade (Clade C), four *Heuchera* species (five accessions) formed a strongly supported clade (BS/PP = 100/1) sister to *M. diphylla*. Hence, while monophyletic for nuclear data, plastid data suggest polyphyly of *Tiarella* as well as other genera in the *Heuchera* group.

## Discussion

We recovered multiple instances of strong incongruence between our nuclear and plastid phylogenies. The genera *Tiarella* and *Heuchera* together with *Bensoniella* were monophyletic in nrDNA data. However, both *Heuchera* and *Tiarella* were clearly polyphyletic in plastid data. Evolutionary processes including convergent evolution, lineage sorting and reticulate evolution could explain discordances between nuclear and plastid phylogenies (Acosta and Premoli, 2010). However, in this case, the probability of generating sequence convergence for an entire plastome is low given the size of the plastid genome and that the sampling locations of the taxa included here are widely geographically distant. Incomplete lineage sorting may likewise be excluded for the following reasons: (1) the plastid DNA in the *Heuchera* group is haploid and maternally inherited (Soltis et al., 1990), and hence the effective population size of plastid loci is generally one-quarter of that of nuclear loci (Birky et al., 1983), which means stochastic lineage sorting of an organelle locus is four times faster than that of a nuclear locus (Moore, 1995); and (2) previous application of a coalescent method on the *Heuchera* group demonstrated that incomplete lineage sorting alone does not adequately explain the plastid phylogeny of this group (Folk et al., 2017).

Plastid capture is an evolutionary process through which inter-species hybridization (commonly with subsequent backcrosses) yields a lineage with a novel genomic combination, where the plastome of one species occurs in the nuclear background of another species (Rieseberg and Soltis, 1991). Plastid introgression is generally thought to be more prevalent than nuclear introgression due to its characteristic inheritance, its usually complete lack of recombination, and the low influence of selection on its conserved housekeeping loci (Martinsen et al., 2001; Avise, 2004). Plastid capture, generally attributed to introgression, has been reported in many plant taxa (Tsitrone et al., 2003; Fehrer et al., 2007; Acosta and Premoli, 2010). These evolutionary events have often been accompanied by a clear absence of introgression of biparentally inherited nuclear genes (Soltis and Soltis, 1995; Fehrer et al., 2007), even in broad surveys of genome loci (Folk et al., 2017).

The *Heuchera* group has been cited frequently as a particularly valuable system for studying plastid capture (Folk et al., 2017 and citations therein). Several examples of plastid capture have been documented in this group using cpDNA restriction-site data together with allozyme data (Soltis, 1991), suggesting plastid capture between species of *Heuchera*, as well as between *Tellima* and *Mitella* (Soltis, 1991; Soltis et al., 1991). Significantly, *Tiarella* and some accessions of *Heuchera* are not only very similar in their cpDNAs, but they also hybridize frequently (Spongberg, 1972). One intergeneric hybrid, known in the nursery trade as “×*Heucherella*” (= *Heuchera* × *Tiarella*), is a common ornamental plant. Previous studies likewise revealed the possibility of ancient hybridization (that is, hybridization events ancestral to extant species) and subsequent plastid transfer between *Heuchera* and *Tiarella* (Soltis et al., 1991; Soltis and Kuzoff, 1995; Folk et al., 2017).

The most striking difference between our nuclear and plastid trees involves the phylogenetic position of *Heuchera* and *Tiarella* (Figures 6, 7). In our nrDNA phylogeny (Figure 6), *Heuchera* was monophyletic when including the monotypic genus *Bensoniella* (BS/PP = -/1). This is consistent with Okuyama et al. (2008) from which we got many of our ITS + ETS sequences. However, using sequences from six nuclear loci (including ITS, ETS and four single-copy nuclear genes, *GBSSI-A*, *GBSSI-B*, *GS-II*, *PepCK*) and 39 morphological characters, Folk and Freudenstein (2014) confidently supported the monophyly of *Heuchera* with concatenation and coalescent analyses. The monophyly of *Heuchera* was further confirmed by a subsequent study (Folk et al., 2017), which employed a targeted enrichment approach to generate a ∼400,000 bp dataset of 277 low-copy nuclear loci. *Tiarella* was well supported as monophyletic and a distant relative of *Heuchera* in our nuclear phylogeny (Figure 6), which is also proved by previous studies (Okuyama et al., 2008, 2012; Folk and Freudenstein, 2014; Folk et al., 2017). Our findings, in combination with previous work, strongly suggest that *Heuchera* and *Tiarella* are both monophyletic and not closely related based on evidence from the nuclear genome. Plastid DNA, by contrast, confidently resolves *Tiarella* species as polyphyletic with more derived positions (Figure 7 and Supplementary Figure S2). *T. polyphylla* and *T. trifoliata* formed a clade sister to Clade A, whereas the two *T. cordifolia* individuals were embedded within in Clade A. Reconciling the discordance between plastid and nuclear phylogenies, particularly among *Tiarella* and *Heuchera* species, requires at least two plastid capture events to explain the incongruences we observed, one to explain the more derived position of *T. polyphylla* and *T. trifoliata* in the plastid phylogeny as compared to nuclear data, and a second to explain the embedded position of *T. cordifolia* among *Heuchera* species. We hypothesize that the ancestor of the genus *Tiarella* initially captured the plastid of a *Heuchera* lineage (most likely an ancestral member of Clade A) through an ancient hybridization. Subsequently, *T. cordifolia* captured the plastid genome from a member, again likely ancestral, of *Heuchera* plastid Clade A. Although we have improved sampling at the population level and among outgroups, our results are congruent with previous work by Folk et al. (2017). Interestingly, a single species of *Heuchera* had two widely different plastid phylogenetic placements, indicating the prevalence of chloroplast capture in the group. Two accessions of *H. richardsonii* formed a clade embedded in the second *Heuchera* clade (Clade B, as seen previously), yet the third individual was embedded in the first *Heuchera* clade (Clade A), representing a putatively novel placement. However, this individual may correspond to a cryptic form of the naturally occurring hybrid *H. richardsonii* × *H. americana* L. examined in previous work and also found in chloroplast clade A (Folk et al., 2017; see also Rosendahl et al., 1936). Finally, the *Mitella* species we sampled in phylogenetic analysis were polyphyletic in both nuclear and chloroplast trees, in agreement with previous studies (Okuyama et al., 2008, 2012; Deng et al., 2015; Folk et al., 2017). However, *M. stauropetala*, was recovered as sister to *Tiarella* in nrDNA data, but was embedded in the first *Heuchera* clade (Clade A) in plastid phylogenies. This inconsistency, as observed in previous work, is also most likely due to the presence of plastid capture.

## Conclusion

In this study, seven species of Saxifragaceae were selected for genome skimming and assembly of complete plastomes, nrITS and nrETS sequences. All the plastomes we sequenced ranged from 154,407 to 155,400 bp in length, encoding 113 identical genes including 79 protein-coding genes, 30 tRNA genes and four rRNA genes. Comparative analysis of plastomes with other Saxifragaceae species revealed that there no rearrangements occurred in gene organization, size variations of the plastomes are purely ascribed to the length differences of LSC, SSC, and IRs regions. Phylogenetic analyses inferred by nuclear and plastid data fully resolved the phylogeny of *Tiarella*, the topology of [(*T. polyphylla*, *T. trifoliata*), *T. cordifolia*] was favored with maximal support (BS = 100%, PP = 1.00). Comparisons of nuclear and plastid phylogenies revealed that plastid capture events have occurred multiple times among species and genera of the *Heuchera* group through ancient hybridization. Furthermore, molecular markers, including plastid hotspots and nuclear polymorphic nSSRs, were efficiently generated for the genus *Tiarella*, for future population genetics and phylogeographic studies.

## Data Availability Statement

The datasets generated for this study can be found in the all the plastome, ETS and ITS sequences sequenced in this study were deposited in GenBank, and the corresponding accession numbers are presented in the manuscript.

## Author Contributions

L-XL, F-DS, and PL conceived the ideas. L-XL and PL contributed to the sampling. L-XL and Y-XD performed the experiment. L-XL and S-YW analyzed the data. The manuscript was written and improved by L-XL, RF, DS, and PL.

## Conflict of Interest

The authors declare that the research was conducted in the absence of any commercial or financial relationships that could be construed as a potential conflict of interest.

## Supplementary Material

FIGURE S1 Visualization of alignment of 17 Saxifragaceae chloroplast genome sequences with *Saxifraga stolonifera* as the reference. The horizontal axis indicates the coordinates within the chloroplast genome. The vertical scale indicates the percentage of identity, ranging from 50 to 100%. Genome regions are color codes as protein coding, intron, mRNA, and conserved non-coding sequences (CNS).

FIGURE S2 Phylogenetic tree reconstruction using maximum likelihood (ML) based on 77 shared protein-coding genes. The inset topology in the upper left shows the relative branch lengths in per-site substitutions. Numbers above the branches represent ML bootstrap/Bayesian posterior probability (BS/PP). Hyphen indicate a bootstrap value or posterior probability <50%.

TABLE S1 Locality and voucher information for seven Saxifrageae species including 11 individuals used in this study. Voucher specimens are deposited at the herbarium of Zhejiang University (HZU), Hangzhou, Zhejiang, China.

TABLE S2 GenBank number for ETS and ITS sequences used in this study. Asterisk indicates the newly generated sequences.

TABLE S3 The detail information of polymorphic nSSRs identified and primer pairs designed for each detected loci within *Tiarella*.
