# Supplementary figures and images for: Plastome Evolution in Saxifragaceae and Multiple Plastid Capture Events Involving Heuchera and Tiarella
Source: Front Plant Sci. 2020 Apr 24;11:361. doi: 10.3389/fpls.2020.00361 (PMC7193090; doi:10.3389/fpls.2020.00361)

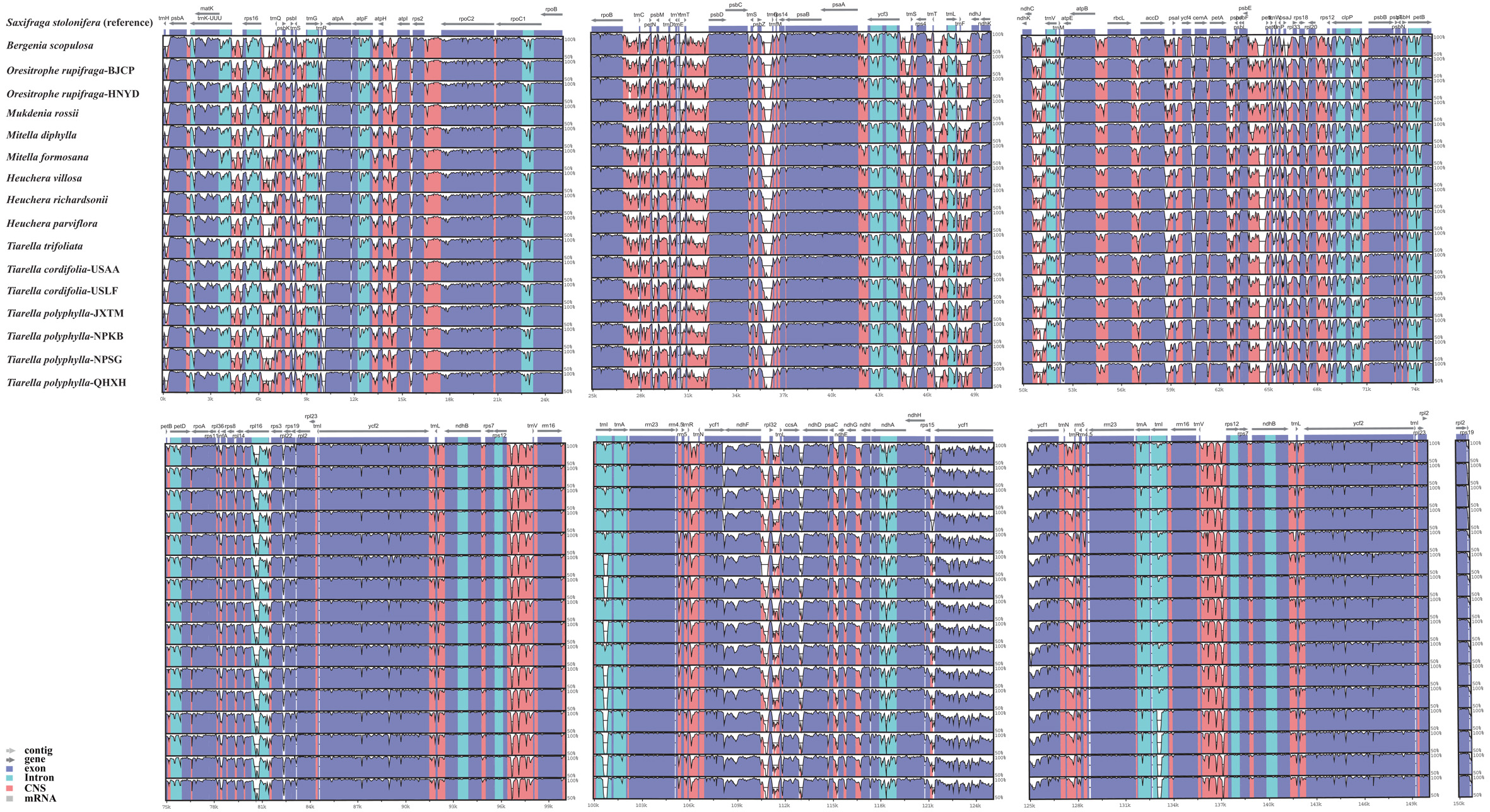

Supplement: FIGURE S1 — Visualization of alignment of 17 Saxifragaceae chloroplast genome sequences with Saxifraga stolonifera as the reference. The horizontal axis indicates the coordinates within the chloroplast genome. The vertical scale indicates the percentage of identity, ranging from 50 to 100%. Genome regions are color codes as protein coding, intron, mRNA, and conserved non-coding sequences (CNS). [file Image_1.JPEG]

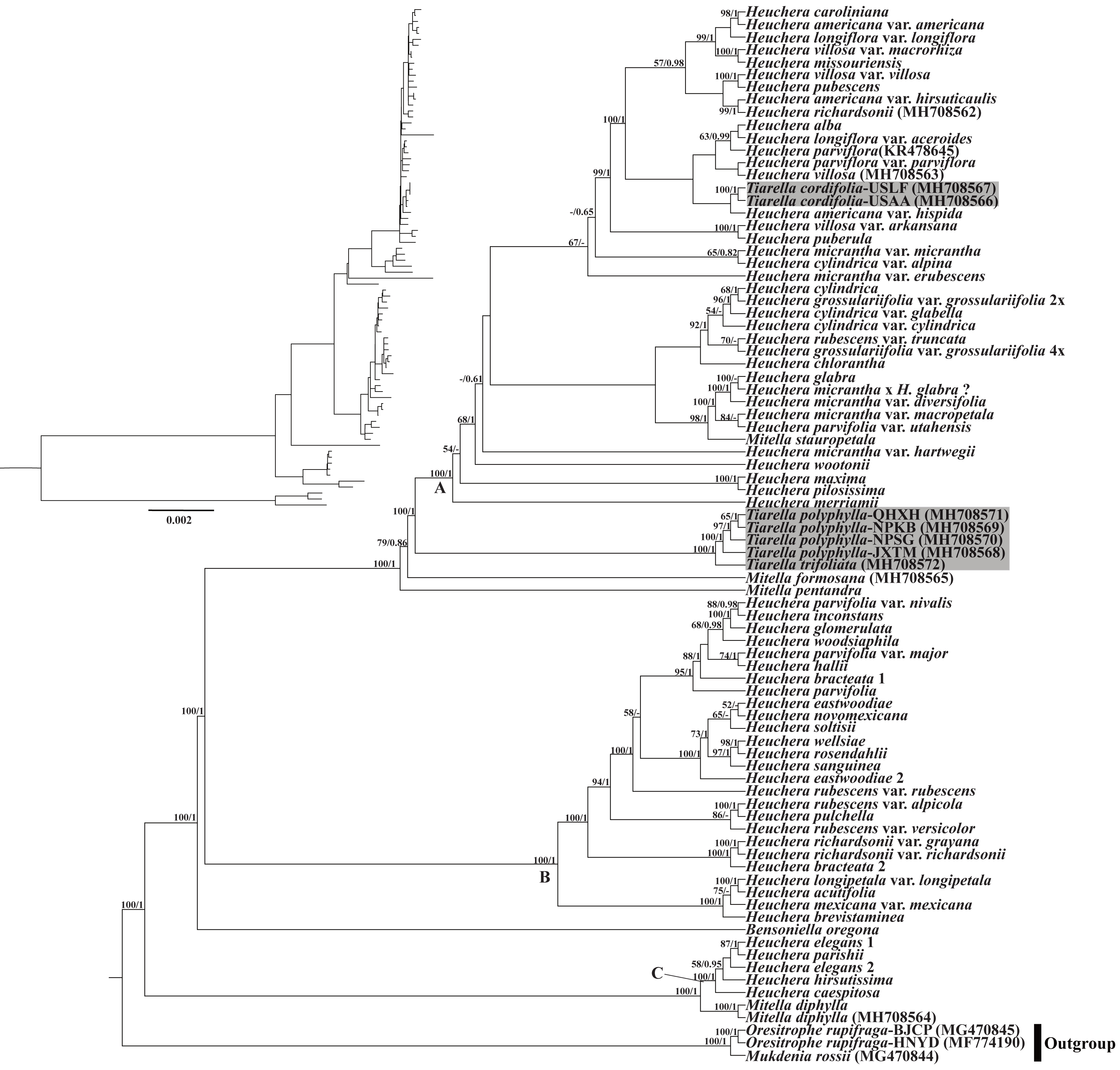

Supplement: FIGURE S2 — Phylogenetic tree reconstruction using maximum likelihood (ML) based on 77 shared protein-coding genes. The inset topology in the upper left shows the relative branch lengths in per-site substitutions. Numbers above the branches represent ML bootstrap/Bayesian posterior probability (BS/PP). Hyphen indicate a bootstrap value or posterior probability <50%. [file Image_2.jpg]
