# Supplementary material for: Plastome Evolution in Saxifragaceae and Multiple Plastid Capture Events Involving Heuchera and Tiarella
Source: Front Plant Sci. 2020 Apr 24;11:361. doi: 10.3389/fpls.2020.00361 (PMC7193090; doi:10.3389/fpls.2020.00361)
Supplement: TABLE S1 — Locality and voucher information for seven Saxifrageae species including 11 individuals used in this study. Voucher specimens are deposited at the herbarium of Zhejiang University (HZU), Hangzhou, Zhejiang, China. [file Table_1.DOC]

Table S1. Locality and voucher information for seven Saxifrageae species including 11 individulas used in this study. Voucher specimens are deposited at the herbarium of Zhejiang University (HZU), Hangzhou, Zhejiang, China.

| Species | Sample code | Voucher no. | Locality | Geographic coordinates | Altitude (m) |
| --- | --- | --- | --- | --- | --- |
| *Tiarella polyphylla* | JXTM | Pan Li LP161083 (HZU) | Huanggangshan, Jiangxi, China | 27°49’32.68" N, 117°43’21.97" E | 1282 |
|  | QHXH | Pan Li LP150221 (HZU) | Mengda, Qinghai, China | 35°47’22.05" N, 102°40’30.04" E | 2577 |
|  | NPKB | T. Hoshino 9668027 (TI) | Kaski, Gandaki, Nepal | 28°22’04.99" N, 83°44’59.68" E | 2200 |
|  | NPSG | H. Ohashi 774114 (TI) | Surke Pati, Nepal | 29°03’02.66" N, 110°28’58.15" E | 2800 |
| *Tiarella cordifolia* | USAA | Pan Li LP162504 (HZU) | Abbeville, Alabama, USA | 29°58’32.16" N, 92°08’03.12" W | 11 |
|  | USLF | Pan Li LP161925 (HZU) | Tompkins, New York, USA | 42°24’04.32" N, 76°35’04.56" W | 290 |
| *Tiarella trifoliata* | - | Pan Li LP174483 (HZU) | UC Botanical Garden, Berkeley, USA | 37°52’18.48" N, 122°16’22.44" W | 54 |
| *Mitella diphylla* | - | Shenyi Wang SY170139 (WIS) | Sauk, Wisconsin, USA | 43°23’09.00" N, 89°47’55.00" W | 296 |
| *Mitella formosana* | - | Pan Li LP151019 (HZU) | Nantou, Taiwan, China | 24°10’52.68" N, 121°16’52.68" E | 3405 |
| *Heuchera villosa* | - | Pan Li LP162370 (HZU) | Carter, Tennessee, USA | 35°50’13.20" N, 83°13’51.96" W | 403 |
| *Heuchera richardsonii* | - | Pan Li LP1005044 (HZU) | Madison, Wisconsin, USA | 43°05’19.16" N, 89°26’22.70" W | 264 |

- Without sample code, because only a single individual was selected in this study.
