# Supplementary material for: Plastome Evolution in Saxifragaceae and Multiple Plastid Capture Events Involving Heuchera and Tiarella
Source: Front Plant Sci. 2020 Apr 24;11:361. doi: 10.3389/fpls.2020.00361 (PMC7193090; doi:10.3389/fpls.2020.00361)
Supplement: TABLE S2 — GenBank number for ETS and ITS sequences used in this study. Asterisk indicates the newly generated sequences. [file Table_2.DOCX]

Table S2. GenBank number for ETS and ITS sequences used in this study. Asterisk indicates the newly generated sequences.

| Species | ETS | ITS |
| --- | --- | --- |
| *Heuchera pubescens* Folk 96-2 | KJ828746 | KJ959298 |
| *Heuchera longiflora* Folk 98-C | KJ828748 | KJ959279 |
| *Heuchera longiflora* Folk 65-C | KJ828740 | KJ959282 |
| *Heuchera americana* var. *americana* Folk I-70 | KJ828789 | KJ959300 |
| *Heuchera richardsonii* Folk I-58 | KJ828786 | KJ959251 |
| *Heuchera richardsonii* Folk 105-1 | KJ828750 | KJ959247 |
| *Heuchera alba* Folk 63-1 | KJ828739 | KJ959296 |
| *Heuchera merriamii* Folk I-63 | KJ828787 | KJ959294 |
| *Heuchera novamexicana* Folk 33 | KJ828722 | KJ959289 |
| *Heuchera eastwoodiae* Folk 35 | KJ828723 | KJ959240 |
| *Heuchera glomerulata* Folk I-5 | KJ828774 | KJ959272 |
| *Heuchera parvifolia* var. *major* Folk 53-1 | KJ828734 | KJ959303 |
| *Heuchera wootonii* Folk 22-1 | KJ828717 | KJ959268 |
| *Heuchera parvifolia* var. *utahensis* Folk I-56 | KJ828785 | KJ959255 |
| *Heuchera grossulariifolia* var. *grossulariifolia* | KJ828767 | KJ959232 |
| *Heuchera rubescens* var. *rubescens* Folk I-19 | KJ828779 | KJ959285 |
| *Heuchera caespitosa* Folk 48-C | KJ828729 | KJ959290 |
| *Heuchera brevistaminea* Folk 37-1 | KJ828724 | KJ959244 |
| *Heuchera rubescens* var. *alpicola* Folk I-43 | KJ828782 | KJ959250 |
| *Heuchera rosendahlii* Folk I-9-1 | KJ828776 | KJ959273 |
| *Heuchera wellsiae* Folk I-7-1 | KJ828775 | KJ959260 |
| *Heuchera rubescens* var. *versicolor* Folk I-15 | KJ828777 | KJ959239 |
| *Heuchera elegans* Folk 44-1 | KJ828727 | KJ959233 |
| *Heuchera sanguinea* Folk I-4 | KJ828773 | KJ959297 |
| *Heuchera woodsiaphila* Folk 23-1 | KJ828718 | KJ959266 |
| *Heuchera acutifolia* Folk I-74-1 | KJ828790 | KJ959242 |
| *Heuchera elegans* Folk I-41 | KJ828781 | KJ959283 |
| *Heuchera longipetala* var. *longipetala* Folk I-21 | KJ828780 | KJ959278 |
| *Heuchera parishii* Folk 43-1 | KJ828726 | KJ959291 |
| *Heuchera pulchella* Folk 20-1 | KJ828716 | KJ959267 |
| *Heuchera mexicana* var. *mexicana* Folk I-51 | KJ828784 | KJ959249 |
| *Heuchera bracteata* Folk 52-1 | KJ828733 | KJ959241 |
| *Heuchera hallii* Folk 58-1 | KJ828737 | KJ959262 |
| *Bensoniella oregona* Folk 148-1 | KJ828763 | KJ959304 |
| *Heuchera glabra* Folk 137-1 | KJ828756 | KJ959265 |
| *Heuchera cylindrica* var. *glabella* Folk 115-1 | KJ828752 | KJ959263 |
| *Heuchera cylindrica* var. *alpina* Folk 151-1 | KJ828764 | KJ959277 |
| *Heuchera cylindrica* Folk 125 | KJ828755 | KJ959275 |
| *Heuchera chlorantha* Folk 138-1 | KJ828757 | KJ959258 |
| *Heuchera cylindrica* var. *alpina* Folk 152-P | KJ828765 | KJ959299 |
| *Heuchera cylindrica* var. *cylindrica* Folk 122-1 | KJ828754 | KJ959295 |
| *Heuchera micrantha* var. *micrantha* Folk 141-1 | KJ828760 | KJ959248 |
| *Heuchera pilosissima* Folk 144-1 | KJ828761 | KJ959293 |
| *Heuchera micrantha* var. *erubescens* Folk 49 | KJ828730 | KJ959246 |
| *Heuchera micrantha* var. *macropetala* Folk 146 | KJ828762 | KJ959252 |
| *Heuchera maxima* | AB292000 | AF158956 |
| *Heuchera villosa* var. *villosa* Folk I-1 | KJ828772 | KJ959271 |
| *Heuchera puberula* Folk 185 | KM495990 | KM496211 |
| *Heuchera parviflora* Folk 229 | KM495992 | KM496213 |
| *Heuchera parviflora* Folk 212 | KM496002 | KM496191 |
| *Heuchera missouriensis* Folk 217 | KM495998 | KM496215 |
| *Heuchera villosa* var. *arkansana* Folk 192-1 | KJ828770 | KJ959274 |
| *Mitella diphylla* | AB248777 | AB248860 |
| *Mitella stauropetala* | AB248780 | AB248863 |
| *Mitella formosana* | *MK056298 | *MK056296 |
| *Heuchera richardsonii* | *MK056302 | *MK056300 |
| *Heuchera villosa* | *MK056301 | *MK056299 |
| *Mitella diphylla* | *MK056297 | *MK056295 |
| *Tiarella polyphylla*-QHXH | *MK056289 | *MK056282 |
| *Tiarella polyphylla*-NPKB | *MK056287 | *MK056280 |
| *Tiarella polyphylla*-JXTM | *MK056286 | *MK056279 |
| *Tiarella polyphylla*-NPSG | *MK056288 | *MK056281 |
| *Tiarella trifoliata* | *MK056290 | *MK056283 |
| *Tiarella cordifolia*-USLF | *MK056285 | *MK056278 |
| *Tiarella cordifolia*-USAA | *MK056284 | *MK056277 |
| *Oresitrophe rupifraga*-BJCP | *MK056293 | *MK056291 |
| *Oresitrophe rupifraga*-HNYD | *MK056294 | *MK056292 |
| *Mukdenia rossii* | *MK056304 | *MK056303 |
